# Ethnomedicinal landscape: distribution of used medicinal plant species in Nepal

**DOI:** 10.1186/s13002-022-00531-x

**Published:** 2022-04-18

**Authors:** Ripu M. Kunwar, Bikash Baral, Sanjeev Luintel, Yadav Uprety, Ram C. Poudel, Binaya Adhikari, Yagya P. Adhikari, Suresh C. Subedi, Chandra K. Subedi, Prakash Poudel, Hem R. Paudel, Basanta Paudel, Laxmi Mahat Kunwar, Kul S. Upadhayaya, Shandesh Bhattarai, Dipesh Pyakurel, Durga H. Kutal, Pramod Pandey, Ananta Bhandari, Gokarna J. Thapa, Narel Y. Paniagua Zambrana, Rainer W. Bussmann

**Affiliations:** 1 Ethnobotanical Society of Nepal, Kathmandu, Nepal; 2 University of Turku, Turku, Finland; 3 Amrit Science College, Tribhuvan University, Kathmandu, Nepal; 4 Central Department of Botany, Tribhuvan University, Kathmandu, Nepal; 5 Nepal Academy of Science and Technology, Lalitpur, Nepal; 6 Institute of Forestry, Tribhuvan University, Pokhara, Nepal; 7 BayCEER,, University of Bayreuth, Bayreuth, Germany; 8 Department of Biology, Arkansas Tech University, Russellville, AR USA; 9 Gandaki University, Pokhara, Nepal; 10 National Herbarium and Plant Laboratories (KATH), Lalitpur, Nepal; 11 School of Environmental Science and Management, Kathmandu, Nepal; 12 Department of Anthropology, Tribhuvan University, Kathmandu, Nepal; 13 Resources Himalaya Foundation, Lalitpur, Nepal; 14 University of Wisconsin-Whitewater, Whitewater, WI USA; 15 Florida Institute of Technology, Melbourne, FL USA; 16 WWF Nepal, Baluwatar, Kathmandu, Nepal; 17 Institute for Biodiversity and Ecosystem Dynamics, University of Amsterdam, Amsterdam, The Netherlands; 18 Department of Ethnobotany, Institute of Botany, School of Natural Sciences and Medicine, Ilia State University, Tbilisi, Georgia

**Keywords:** Medicinal plants, Distribution, Traditional medicine, Culture, Heatmaps, Mountains

## Abstract

**Background:**

The risk of losing traditional knowledge of medicinal plants and their use and conservation is very high. Documenting knowledge on distribution and use of medicinal plants by different ethnic groups and at spatial scale on a single platform is important from a conservation planning and management perspective. The sustainable use, continuous practice, and safeguarding of traditional knowledge are essential. Communication of such knowledge among scientists and policy makers at local and global level is equally important, as the available information at present is limited and scattered in Nepal.

**Methods:**

In this paper, we aimed to address these shortcomings by cataloguing medicinal plants used by indigenous ethnic groups in Nepal through a systematic review of over 275 pertinent publications published between 1975 and July 2021. The review was complemented by field visits made in 21 districts. We determined the ethnomedicinal plants hotspots across the country and depicted them in heatmaps.

**Results:**

The heatmaps show spatial hotspots and sites of poor ethnomedicinal plant use documentation, which is useful for evaluating the interaction of geographical and ethnobotanical variables. Mid-hills and mountainous areas of Nepal hold the highest number of medicinal plant species in use, which could be possibly associated with the presence of higher human population and diverse ethnic groups in these areas.

**Conclusion:**

Given the increasing concern about losing medicinal plants due to changing ecological, social, and climatic conditions, the results of this paper may be important for better understanding of how medicinal plants in use are distributed across the country and often linked to specific ethnic groups.

**Supplementary Information:**

The online version contains supplementary material available at 10.1186/s13002-022-00531-x.

## Introduction

Catalogues have recorded 1515 to 2331 useful medicinal and aromatic plants in Nepal [1–7], reporting their importance in alleviating human suffering. Medicinal and aromatic plants have long been used in Nepal for subsistence, household economy [8], and traditional medicines [9]. These plants are also important for income generation [10], and they do support market economy [11]. The use of medicinal plants is supported by countries’ rich biodiversity [12], over 125 ethnic groups [13], and five disparate physiographic regions [14, 15]. In the face of climate change [16], land use change [17], outmigration, and sociocultural and economic transformations [18], the tradition of medicinal plant collection and use in remote and rural areas has undergone significant changes in the recent decades [19, 20]. Following predicted climate change, the population of medicinal plants is likely threatened, and the suitable habitat range for medicinal plants is likely to be shrink, resulting in significant alteration of traditional collection sites and practices [21–23]. Thus, changing ecological and social conditions has transformed and shaped traditional system and knowledge of medicinal plant use in Nepal to match the new circumstances [22, 24].

Therapeutic efficacy [25], and geography [26–29] are other underlying factors that influence the collection and use of medicinal plants in traditional medicine systems. Geographic isolation has strengthened the indigenous knowledge of plant use [30–32]. Human communities inhabiting remote and rugged ecosystems adopt diverse livelihood strategies such as collecting and utilizing locally available medicinal plants, summer grazing, animal husbandry, and so on [19, 33, 34]. The adopted strategies are characterized by altitudinal gradient, distance, and accessibility [35]. Nearby habitats and easily available plants have frequently been accessed for collection [28, 36]. In the context of declining pattern of sharing of traditional knowledge complicated by outmigration, independent use [37] and adaptation to local environment through experimentation have long been established [38–40]. Thus, we would expect that medicinal plant use is patterned by spatial features [32].

Medicinal plant species richness in Nepal peaks between 1000 and 2500 m asl [1–3, 41] and then decreases with elevation [42]. This indicates the outstanding contribution of Mid-hills and lower mountain physiography to medicinal plant diversity [19, 43]. Out of many, elevation and type of ecosystem could be the factors for species distribution inferring that the larger the area, the more the number of species is [44, 45]. Because, according to habitat heterogeneity hypothesis, large areas contain more species due to more available niches [46, 47]. If the non-random selection of plants is underlying mechanism directing choice [25], then the number of plants collected and used in a site should correlate with the number of available species [48].

There are some studies in Nepal describing distribution and uses of ethnomedical plants [2–4, 32, 41, 42], however, the accounts of medicinal plants with interactions of spatial and cultural variables remain under-explained. Yet, it remains unclear how geography and cultures with plant use traditions reflect in medicinal plant distribution, especially for the country where medicinal plant collection, trade, and use, are the major source of livelihood. To be precise, the analyses of association between geographical attributes and the number of medicinal plants uses are particularly limited in the Himalaya [49]. Understanding distribution pattern and selection of medicinal plants for local use may aid in identifying critical resource sites and hotspots in the region, contributing to plant biodiversity conservation. Heatmaps are used to easily identify clusters (hotspots) that can be used to map the density of medicinal plants [50, 51] and to devise the management strategies. So far, little attention has been received in management of medicinal plant in the Nepal Himalaya [52], which is extremely vulnerable to climate change and is being affected by rapid land-use change and growing anthropogenic pressures [17, 53, 54]. As part of a conservation effort, it is critical to catalogue the dynamics of medicinal plants, including their distribution, indigenous usage, and management [55]. The relationship between humans and plants, and consequently selection of plants for a use, can be seen as a complex set of interactions based on socio-culture [26, 56, 57], plant species availability, and treatment need, resulting in a 'herbal landscape' [58].

In this regard, we focused our analysis specifically on i) spatial distribution of medicinal plants used by ethnic groups in Nepal, ii) heatmaps revealing hotspots of total medicinal plant species, high trade-value medicinal plant species and aromatic medicinal plant species that are being used in Nepal, and iii) relationship between medicinal plants and their uses with geographical and cultural factors. We hypothesized that use of medicinal plants in Nepal is influenced by geographical and cultural factors, and follow spatial patterns. In this paper, we considered medicinal plants as those plants that have long been used in traditional pharmacopoeias. They are used as an alternative and/or as complementary medicine. Furthermore, we separated medicinal plants into traded medicinal plants, which are defined as plants traded to pharmaceuticals, nutritional supplement products, natural health products, cosmetics and other personal care products, and culinary products [59], based on the explanation made by Medicinal Plants Specialist Group (2007). Aromatic plants were sorted out following Gurung and Pyakurel [60].

## Materials and methods

### Study area description, data collection and verification

Politically, Nepal is divided into seven provinces, 77 districts, and 753 local bodies [61]. Longitudinally, the country has three distinct regions, Western Nepal (from 80° E to 83° E), Central Nepal (from 83° E to 86° 30′ E), and Eastern Nepal (from 86° 30′ E to 88° 12′) [62]. The country has five disparate vertical physiographic regions from south to north: (i) Tarai (< 500 m above sea level) with 14% area, (ii) Siwalik (500–1000 m asl) with 12% area; (iii) Mid-hills (1000–3000 m asl) with 30% area; (iv) high mountain (3000–5000 m asl) with 20% area; and (v) High Himalaya (above 5000 m asl) with 24% area [14, 15]. Owing to varied geography, the country has over 13000 species of plants [63], including about 7000 species of flowering plants [64, 65].

Relevant literature published between 1975 and July 2021 and described the uses of medicinal plants of particular ethnic groups in a particular district or a village were reviewed. The general ethnobotanical publications without the district or village level references were excluded. Data were collected from research journal articles (searched in Google Scholar) as well as a limited number of ‘gray’ literatures. The ‘gray’ literature (university theses and government reports that account the use reports of medicinal plants at district or village level) only passed through the external reviews, and multilevel consultations were considered for systematic review [66]. Information obtained from the review was verified and cross-checked in the fieldwork.

**Fig. 1:**
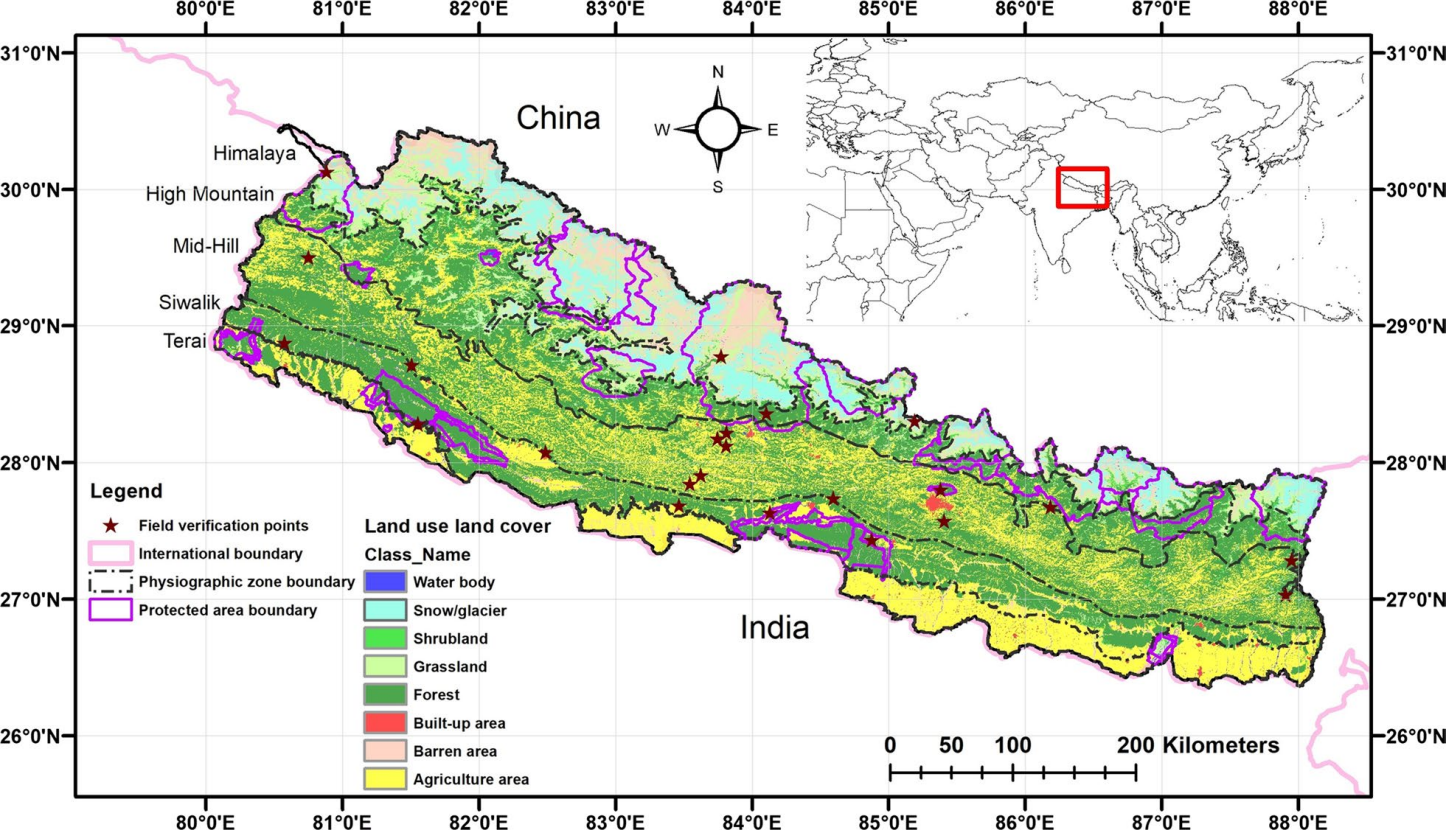
Study area map showing protected areas and physiography of the country and field verification points

A total of 23 fieldworks were made between 2017 and 2020 by the authors RMK, RCP, SB, SL, YA, and YU in 21 randomly selected districts across the country (Fig. 1). There were three, six, and fourteen fieldworks from Eastern, Western, and Central Nepal, respectively, with the majority of fieldworks concentrated in the Mid-hill districts (11) followed by Tarai Siwalik districts (eight) and mountainous Himalaya (four). All the records were compiled together in a single database, and cross-checked for validation for their authenticity and duplication by the second, third, and fourth authors. Plant taxon was verified by using the plant list website www.theplantlist.org.

### Data analysis

After systematic review of over 275 pertinent literatures describing ethnomedicine of Nepal, we separately catalogued the number of medicinal plant species, traded medicinal plant species, and aromatic medicinal plant species used by ethnic groups in a particular district for the whole country. For each district, mean elevation, total area, human population, human density, and ethnic groups were tabulated for analysis. We entered the acquired bibliographic data into a MS Excel file, noting the plant species name, local name, family name, medicinal use, plant parts used, ethnic group, the district from where the use was reported, and the reported year [67]. We referred to the national census data [13] for population, composition, and distribution of ethnic groups by districts. For further interpretation, we used a verification method, which allows description of both quantitative and qualitative data and information at temporal (year), spatial (district), cultural (ethnic group), and species (total medicinal plants, traded medicinal plants, and aromatic medicinal plants) levels.

A binary index (1 for the presence of a use record of a particular species in the district, and 0 for none) was employed to calculate the total record and use of medicinal plants in each district for the heatmap generation. The inverse distance weighing (IDW) was used to interpolate unsampled regions to prepare heatmaps [68]. IDW interpolator assumes that the local influence that each point possesses diminishes with distance, resulting in the decreasing influence of the variable with increasing distance from its sampled location [69]. The IDW is estimated through moving average technique generating a value that is less than the local maximum and greater than the local minimum [69, 70] attributing greater weight to the points that are closer to the processing cell that those which are further away (Eq. 1).

1 $${z}_{j}=\frac{{\Sigma }_{i}\frac{{z}_{i}}{{d}_{ij}^{n}}}{{\Sigma }_{i}\frac{1}{{d}_{ij}^{n}}}$$

In Eq. 1, estimated value for unknown point at location j is represented by *Z*_*j*_, *d*_*ij*_ is distance between point *i* to unknown point *j*, *Z*_*i*_ is value of a known point *i*, whereas *n* is user defined exponent of distance (taken as 2 in this case, default value). The in-point features were represented by 77 districts and the *z* field represented numerical values (number of medicinal plant species used in a whole, number of aromatic medicinal plant species and number of traded medicinal plant species) corresponding to each district. We used variable search radius, where the number of points was set to 12 (default value) and the maximum distance to limit the nearest search sample was also the default value (the length of the extent's diagonal). The procedure of interpolation was carried out using the spatial analysis technique of GIS (ArcGIS 10.7).

Although heatmaps provide useful information on spatial patterns, statistical tests would be essential to interpret the significant differences among the variables [68]. We used generalized linear model regression to find the relationships between district area, human population, human density, elevation, and number of ethnic groups as the independent variables, and number of medicinal plant species in use as the dependent variable. This approach also helped us identify key factors influencing ethnomedicine and geographical areas with data gap (if any), which can support in future research planning for sustainable management of medicinal plants and preservation of ethnomedicinal knowledge. All the analyses were performed in R studio in R 4.1.2 (R Development Core Team 2021).

## Results

We recorded a total of 8737 use accounts of 1762 medicinal plant species from 77 districts in Nepal. This is the first state-of-the-art documentation of over 1700 used medicinal plant species in Nepal with district reference. We found that the number of ethnomedicinal uses varied among the species, family, and district. Of the species, 129 were from Asteraceae, 114 from Fabaceae, 65 from Lamiaceae, 54 from Rosaceae, and 53 from Poaceae. The species, *Centella asiatica* L. (Urb.) (Ghodtapre in Nepali), was used in 57 (74%) districts out of the total 77, followed by *Acorus calamus* L. (Bojho in Nepali) in 56 (72%), *Asparagus racemosus* Willd. (Kurilo in Nepali) in 49 (63%), *Cuscuta reflexa* Roxb. (Akashbeli in Nepali) in 47 (61%), and *Achyranthes aspera* L. (Apamarga in Nepali) in 46 (59%) districts.

**Fig. 2:**
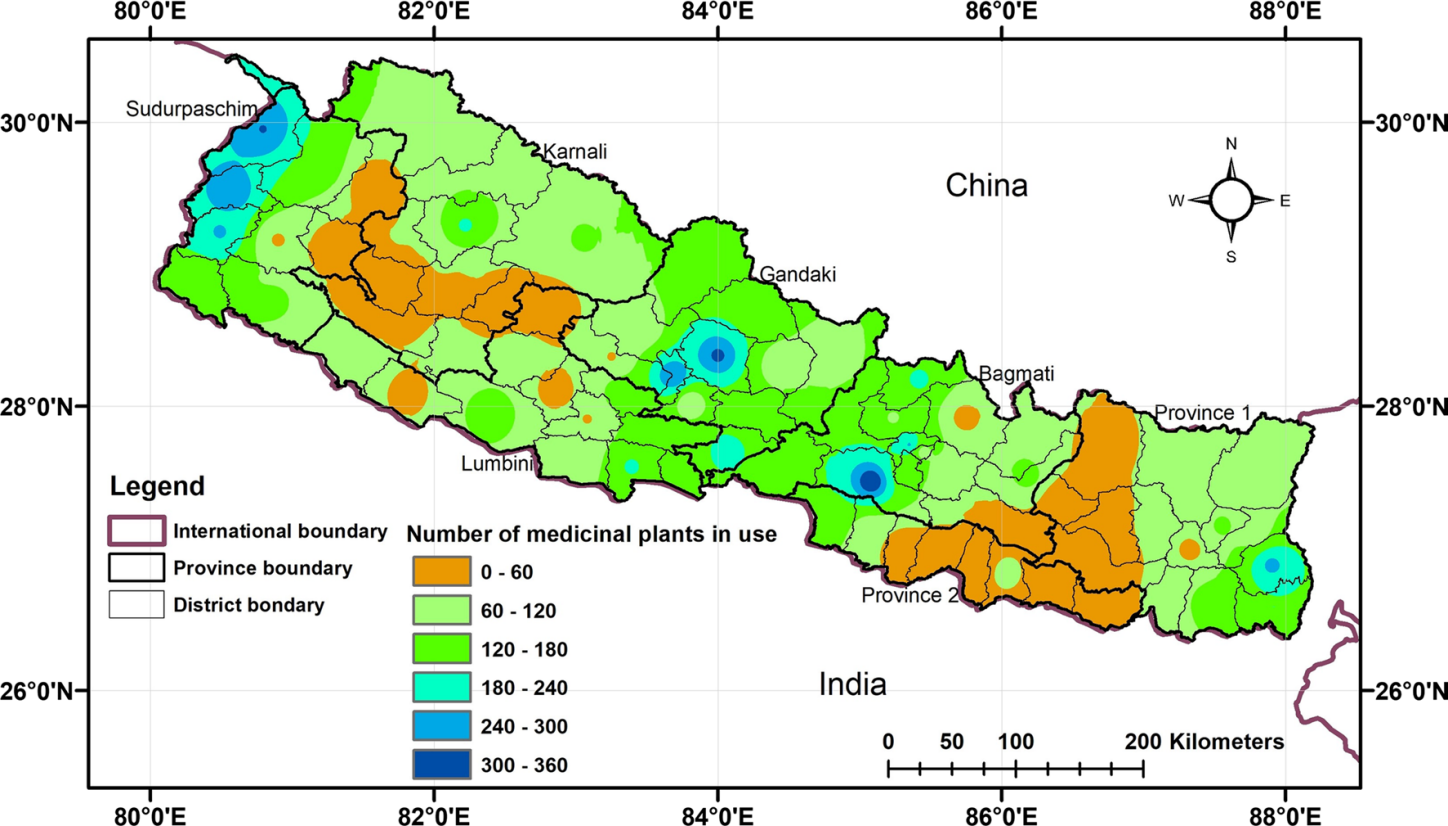
Heatmap depicting the distribution of ethnomedicinal plants. The abundance of medicinal plants used as hotspots is represented in blue color, whereas the lowest abundance is represented by orange

A varied account of used medicinal plant species was also observed at district level: Makawanpur district (359 species, 20%), Kaski (315, 18%), Parbat (301, 17%), Darchula (300, 17%) and Ilam (290, 16%) having the most, and Saptari (1 species), Siraha (2), Rautahat (2), Udayapur (2), Khotang (2), and Dailekh (2 species) the lowest (Fig. 2, Additional file 1). It is noted that the highest number of used medicinal plant species was recorded from Makawanpur, Kaski, and Parbat districts from central Nepal, and Darchula and Ilam from Western and Eastern Nepal, respectively. Makawanpur and Ilam districts constitute Mid-hill physiography, whereas Kaski, Parbat, and Darchula districts exemplify Mid-hills and mountainous region. However, we found no significant association between district area and number of used medicinal plants (*F* = 0.01, *t* = 0.11, *p* = 0.91). The total number of medicinal plant species in use was also assessed against districts’ human population, density, and ethnic group count, yielding the following results (*F* = 0.63, *t* = 0.79, *p* = 0.42), (*F* = 1.23, *t* = 1.11, *p* = 0.26), and (*F* = 0.35, *t* = 0.59, *p* = 0.55), respectively. Neither of the results was statistically significant (Table 1). Both ethnic counts and human populations were decreased as increasing elevation (ethnicity: t = − 8.257 *p* =  < 0.005, population: t = − 7.06, *p* = 0.005).

**Table 1:** Regression analysis coefficients of independent variables (human population, population density, district area, number of ethnic groups in district, and mean elevation of the district), against dependent variables (used medicinal plants in whole, traded medicinal plants and aromatic medicinal plants) (*significant)

Independent variables	Used medicinal plants (all)			Traded medicinal plants			Aromatic medicinal plants		
	F	t	*p*	F	t	*p*	F	t	*p*
Human population	0.63	0.79	0.42	0.01	0.12	0.89	0.28	− 0.53	0.59
Area	0.01	0.11	0.91	0.18	0.42	0.67	0.56	0.74	0.45
Population density	1.23	1.11	0.26	0.43	0.65	0.51	0.81	0.90	0.37
Number of ethnic groups	0.35	0.59	0.55	0.00	− 0.07	0.94	0.13	− 0.37	0.70
Mean elevation	0.51	0.71	0.47	1.17	1.08	0.28	3.94	1.98	0.05*

Distribution of ethnomedicinal plants was associated with the protected areas such as Api-Nampa Conservation Area of Darchula and Annapurna Conservation Area of Kaski but it did not reflect to other protected areas. The least account was reported from Saptari, Siraha, Rautahat, Udayapur, and Khotang districts in eastern Nepal and Dailekh district in Western Nepal. The three districts (Saptari, Siraha, and Rautahat) are at lowland Tarai of Madhes Province. High-altitude districts (Sindhupalchok, Solukhumbu, Mugu) also possessed less number of medicinal plant species in use. Both the lowland Tarai and high-altitude districts possessed less number of used medicinal plants inferred that elevation does not linearly influence the medicinal plants use (Table 1). The identified ethnomedicinal plant hotspots, colored by dark blue (Fig. 2), occur in the Mid-hills and mountainous regions, which lie between lowland Tarai and the Himalayas. The Mid-Western and Western Nepal hill districts have low records of medicinal plant species in use (orange color, Fig. 2).

**Fig. 3:**
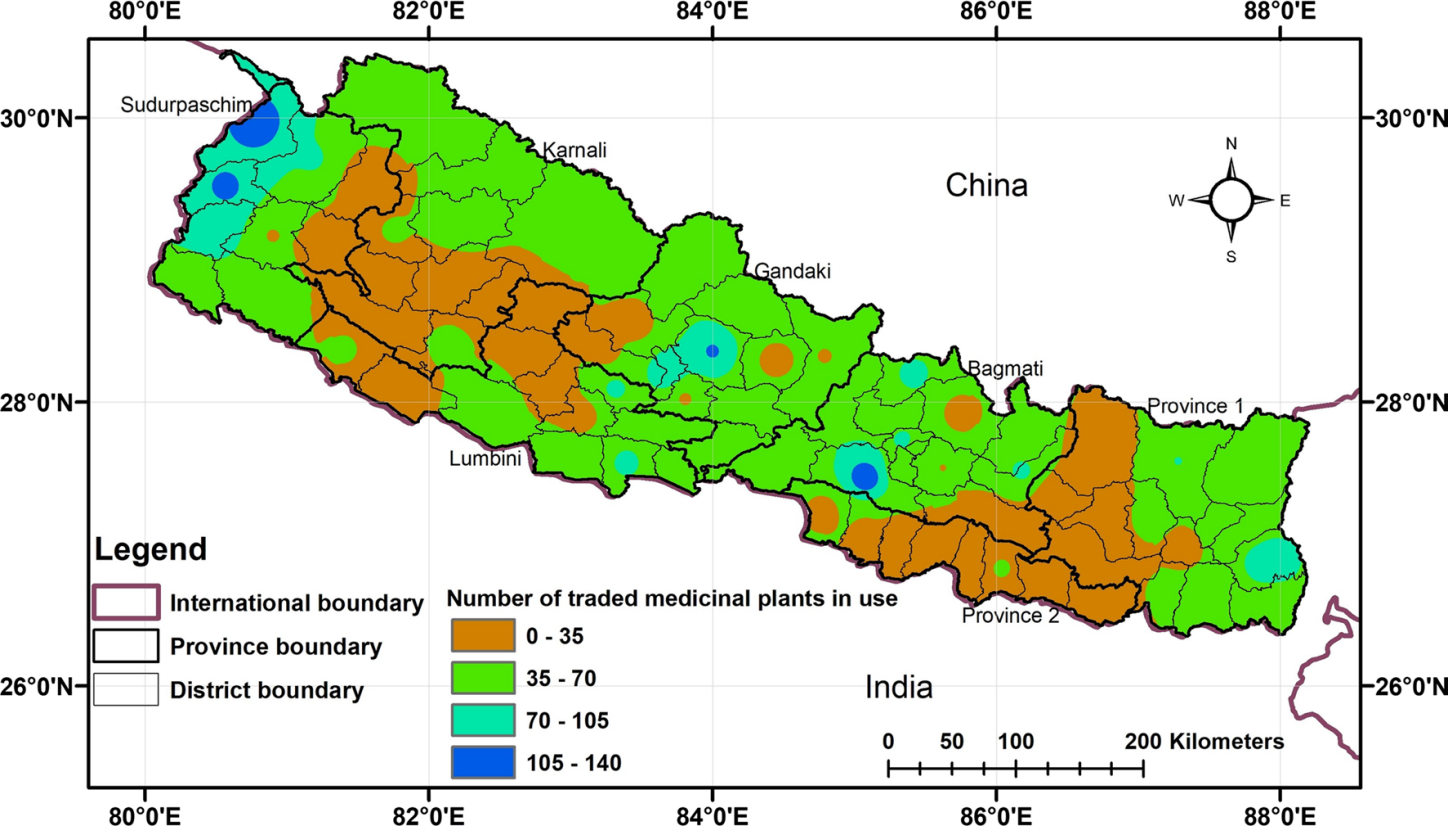
Heatmap depicting the distribution of traded medicinal plants in use

**Fig. 4:**
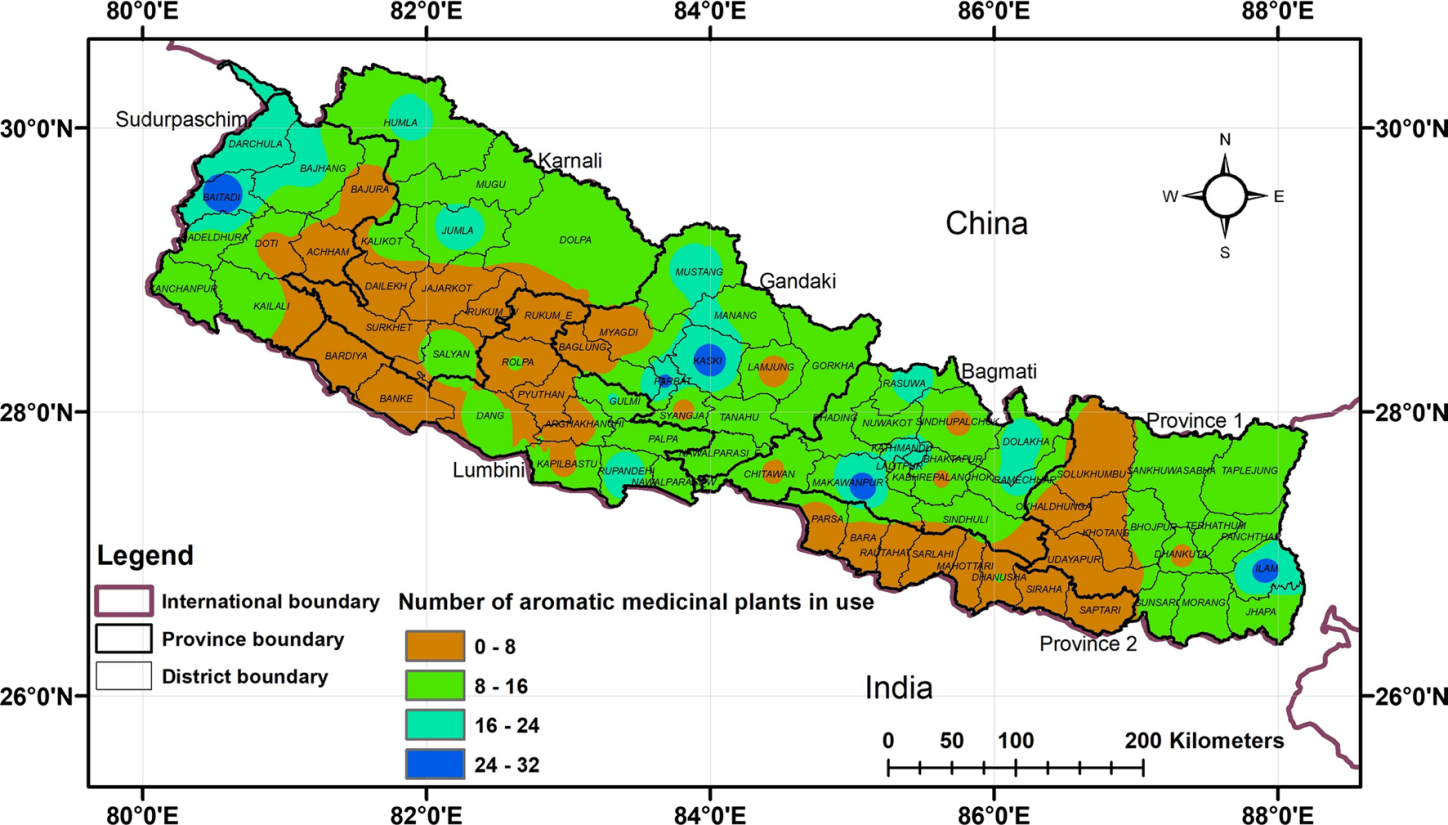
Heatmap depicting distribution of aromatic medicinal plants in use

We recorded 296 traded medicinal plants in Nepal, of which 273 (92%) are being used as ethnomedicine. The hotspots of traded medicinal plant species in ethnomedicinal use were consistent with that of overall ethnomedicinal plants, with highest reports from Makawanpur (137 species), Darchula (125 species), Baitadi (111 species), Ilam (110 species), Kaski (109 species), Dadeldhura (103 species), Parbat (96 species), Bajhang (87 species), and Panchthar (86 species) (Fig. 3, Additional file 1) while the hotspots of medicinally used  aromatic plants are scattered and covered across all physiographic regions of the country (Fig. 4). Among the reported 64 aromatic plants, 49 (76%) are being used for traditional medicine in Nepal. Higher number of aromatic medicinal plant species were found to be used in increasing elevation of the districts.

## Discussion

Our result of distribution of ethnomedicinal plants is based on the limited (~25% out of > 1000 [4]) published ethnobotanical literatures, verified by field visits, data analyses, and mapping. We compared our result with the publicly available medicinal plant database of Nepal and found that there were 80% of 1515 species common [7]. The database including other accounts [1–3, 5, 6] which enumerated the medicinal plants and feebly documented the species uses by districts. The account of medicinal plants and use reports distinctly varied among the species, families, and districts in our study. By family, Asteraceae, Fabaceae, Lamiaceae, Rosaceae, and Poaceae were extensively utilized. Similar accounts of over-utilization of these families is common in both Nepal [9, 26, 71] and elsewhere [71, 72].

The districts with easier accessibility (Makawanpur, Kaski, Parbat, Ilam, Kathmandu) and conservation projects implemented (Baitadi, Darchula, Dadeldhura) were frequently researched [70] resulting in a higher number of ethnomedicinal plant recordings. This suggests that the availability of publications could be linked to utilization of resources. More publications are from the districts in the Western, and Far-Western regions, where active ethnobotanical research programs are linked to established academic institutions like Institute of Forestry (IoF), Institute of Agriculture and Animal Sciences (IAAS) of Tribhuvan University and Kathmandu University. The Kailash Sacred Landscape Conservation and Development Initiative (KSLCDI) is a collaborative project that is being implemented across the borders of China, India, and Nepal (Baitadi, Bajhang, Darchula, and Humla districts) [70] offered a good number of publications of ethnomedicinal accounts [12, 18, 73].

A lower number of medicinal plant species were used at high-altitude districts (such as Sindhupalchok, Solukhumbu, and Mugu) likely to be attributed to low human population, high outmigration, rugged physiography, and limited access including eco-physical constraints [34] due to snow and glaciers. High mountains and Himalaya regions of Nepal constitute over 35% area as snow and glacier cover (Fig. 1). The rugged and undulating ground and alternating peaks and valleys may cause limited access to plant collections resulted in underreporting of usefulness of medicinal plants in such areas. The livelihood at rural, remote, and rugged high-altitude areas is featured with low level of acculturation [74] and defined by local vegetation [75], altitudinal gradient and accessibility [28]. It has been argued that people living in low-acculturation areas may use fewer plant species for medicine [76], which could contribute to the area’s actual low medicinal plant counts. The high-altitude areas are relatively homogenous by ethnic composition. The three districts Dolpa, Jumla, and Manang possess only 19 ethnic groups each and the rest high-altitude districts acquire ethnic groups only between 21 and 52 each out of 125 [13]. Low plant species diversity in high-altitude areas also resulted in limited account of ethnomedicinal plant species being used [77–79]. A limited number of used medicinal plant species were also reported from eastern Tarai, and this could partially be attributed by a low level of medicinal plant diversity [80, 81], limited ethnobotanical research, extensive cover of agricultural land, and rapid land use change [82]. It is noted that the Tarai is one of the less studied areas of Nepal in terms of ethnobotany [83] but it harbors many useful medicinal plant species [84]. Thus the Tarai region may need to explore more on ethnobotanical information.

Four provinces with the highest number of used medicinal plants, the highest in Bagmati province (1955 use records, 22%) and Gandaki province (1511 use records, 17%) followed by Sudurpaschim province (1350 records, 15%), may be due to presence of higher coverage of Mid-hills and mountainous areas which hold the highest number of medicinal plant species in use, and it could be related to their larger human population, associated use, and cultural heritage [85]. The result was substantiated by the findings of positive although insignificant association between human population density and number of used medicinal plant species in the districts (Table 1). The Tarai and Mid-hill regions have the greatest diversity of ecosystems and species in Nepal [86, 87] as it covers 2.25 million hectare forest [80] and contains over two third of the country’s total human population [13]. For example, the most frequently used five species *Centella asiatica* (ranges within altitude 500–2100 m), *Acorus calamus* (100–2300 m), *Asparagus racemosus* (600–2100 m), *Cuscuta reflexa* (1100–3100 m), and *Achyranthes aspera* (100–2900 m) were recorded from lowland and Mid-hills (100–3100 m asl). Accessible lowland and Mid-hills possessed the large number of used plants, whereas a much lower number of useful medicinal plant species were recorded from high-altitude remote districts. Therefore, higher population density areas may lead to higher ethnobotanical usage [1]. However, no association between district area and number of used medicinal plant species implied that the ethnomedicinal account is irrespective to the size of a site rather it is dependent on physiography and human culture. There is a significant positive association between number of ethnic groups and medicinal plant used props that cultural diversity (number of ethnic groups in the district) may have significantly influenced the use of medicinal plant species. The distribution pattern of ethnomedicinal plants also did not correspond well with the cover of protected areas, as observed in earlier studies [2, 3, 15].

Of the recorded 300 traded medicinal plant species in Nepal [42], 273 were being used in ethnomedicine which implies that medicinal plant species are used not only for primary health care, but also for household economy and livelihood. The hotspots of traded medicinal plants were consistent with that of overall ethnomedicinal plants with some outstanding records from Makawanpur, Ilam, Panchthar, Darchula, Baitadi, Kaski, Parbat, and Bajhang districts. Of the districts, Makawanpur is from Central Nepal, Ilam and Panchthar from Eastern Nepal, and the rest from Western Nepal.

Local livelihoods of people of Western Nepal are heavily dependent on the collection, use, and trade of medicinal plants [54, 88, 89]. The Western Nepal and Karnali province hold the significant number of aromatic and medicinal plants in trade [90–92] as the regions have the highest production [93]. Yet, the hotspots of traded medicinal plants in ethnomedicinal use were slightly inconsistent to that of production and trade centers. Charmakar et al. [94] stated that medicinal plant use and trade independently show the insignificant positive association with the production. Some medicinal plants are not utilized as a resource base for traditional medicine, but rather used as a means of collecting for markets and bartering with the people who frequently practice transhumance [95], which led to some inconsistencies. Thus, highly productive areas for medicinal plants may not be always rich in used medicinal plant species as well as for the trade. For instance, high-mountain areas provide resource-base for trade-potential medicinal plants [96], whereas the ethnomedicinal applications of traded medicinal plants are prevalent in the middle mountains [35]. Thus, the rich sites of medicinal plant production, use, and trade are different.

Mid-hills and mountainous districts hold the highest number of medicinal plant species in use, and it may be linked to the larger human population and ethnic groups of that area. This could be associated with human population, cultural heterogeneity, and accessibility as well. Therefore, identifying hotspots of medicinal plants in use and trade would allow deciphering information regarding the centers of plants use, distribution, therapeutics, and trade. It can be argued that human populated and medicinal plant hotspot areas prospect the well-being of local livelihood, market economy, and drug discovery [97].

## Conclusions

This study, which produced heatmaps, and uncovered hotspots of medicinal plants based on ethnomedicinal literatures and field verification, and assessment of interaction of geography, culture and livelihood, is the first state-of-the–art appraisal providing the scopes for conservation of medicinal plant species and their associated knowledge. The developed heatmaps showed spatial hotspots and poor sites of ethnomedicinal plants, which is useful for analysis of geographical and ethnobotanical connections. From this study, we presented distribution of ethnomedicinal plants for the whole country of Nepal as an ethnomedicinal landscape where hotspots are overlaid in the Mid-hills and Mountains. With this study, we contribute to a growing body of literature that, in various ways, argues for the need of integrating the geographical and cultural attributes for ethnobotanical studies, which ultimately helps conserve the ethnomedicinal knowledge and medicinal plant species for long run.

## Supplementary Information

**Additional file 1**. Data table of geography, demography, ethnicity, and used medicinal plants by district

## Data Availability

All relevant data are within the manuscript and its supporting information files.
